# Regional disparities of prostate cancer mortality in Ecuador: an examination of trends and correlates from 2004 to 2019

**DOI:** 10.1186/s12889-023-15941-z

**Published:** 2023-05-29

**Authors:** Katherine Simbaña-Rivera, J. Smith Torres-Roman, Mabel R. Challapa-Mamani, Jhon Guerrero, Gabriel De la Cruz-Ku, Jorge Ybaseta-Medina, José F. Martinez-Herrera

**Affiliations:** 1 Centro de Investigación para la Salud en América Latina (CISeAL), Facultad de Medicina, Pontificia Universidad Católica del Ecuador (PUCE), Quito, Ecuador; 2 Toxicology Unit, Research Institute of Biomedical and Health Sciences (IUIBS), Universidad de Las Palmas de Gran Canaria (ULPGC), Paseo Blas Cabrera s/n, Las Palmas de Gran Canaria, 35016 Spain; 3 Latin American Network for Cancer Research (LAN–CANCER), Lima, Peru; 4 Escuela Profesional de Medicina Humana, Universidad Privada San Juan Bautista, Ica, Filial Chincha Perú; 5 Sociedad Cientifica de Estudiantes de Medicina de la Universidad César Vallejo, Trujillo, Perú; 6 Scientific Association of Medical Students, Universidad Central del Ecuador, Quito, Ecuador; 7 Universidad Cientifica del Sur, Lima, Peru; 8 Universidad Nacional San Luis Gonzaga de Ica, Ica, Peru; 9 Cancer Center, Medical Center American British Cowdray, Mexico City, Mexico

**Keywords:** Prostatic neoplasms, Trends, Mortality, Spatial analysis, Ecuador

## Abstract

**Background:**

Prostate cancer is the leading cause of cancer death in Ecuadorian men. However, there is a lack of information regarding the evolution of prostate cancer mortality rates in Ecuador and its regions in the last few decades.

**Objective:**

The aim of this study was to report prostate cancer mortality rates in Ecuador and its geographical areas and observe the evolution of these rates between 2004 and 2019.

**Methods:**

An observational ecological study was conducted, analysing data for prostate cancer deaths from 2004 to 2019 in Ecuador. Age standardized mortality rates (ASMR) were calculated per 100,000 men using the world standard population with the direct method proposed by SEGI. Joinpoint regression analysis was performed to examine mortality trends. We used a Cluster Map to explore relationships among regions between 2015 and 2019.

**Results:**

Ecuador reported 13,419 deaths by prostate cancer between 2004 and 2019, with the Coastal region accounting for 49.8% of the total deaths. The mean age at death was 79 years (± 10 years), 91.7% were elderly (more than 65 years old) and had primary education (53%). Deaths by prostate cancer were more frequently reported among mestizos (81.4%). There were no significant variations in these percentages in Ecuador and its regions during the study period. Carchi province had the highest mortality rate in 2005 and 2019 (> 13 deaths per 100,000). Heterogeneity in the evolution of mortality rates was reported among the provinces of Ecuador. Azuay decreased in the first few years, and then increased from 2010 to 2019, whereas Guayas and Pichincha decreased throughout the whole period.

**Conclusion:**

Although prostate cancer mortality rates in Ecuador have remained stable over the past few decades, there are significant disparities among the different regions. These findings suggest the need for the development of national and provincial registration measures, integrated healthcare actions, and targeted interventions to reduce the burden of prostate cancer in the Ecuadorian population.

## Background

In 2020, GLOBOCAN reported approximately 1.4 million new cases (7.3% of all cancer sites) and 375,000 deaths (3.8% of all cancer sites) attributed to prostate cancer, making it the most common malignant neoplasm in men globally [1, 2]. However, incidence and mortality rates for prostate cancer present significant heterogeneity across different regions of the world. While Australia/New Zealand and several European regions have the highest incidence rates (above 75 per 100,000 men), the Caribbean and Middle Africa exhibit the highest mortality rates (above 25 per 100,000 men) [1–3].

Despite GLOBOCAN projections, epidemiological information by country in Latin America remains scarce. During the last decades, the life expectancy of the population of Latin America has increased, together with the increase in aging, thereby shaping current epidemiological profiles [4, 5]. This change inevitably results in an increasing incidence of cancer, including prostate cancer [6, 7], which constitutes one of the major public health problems in Latin America as well as a major challenge for health systems to respond to the growing burden of cancer [5].

In 2020, countries in Latin America, such as Suriname, French Guiana, Venezuela, and Guyana, reported cancer mortality rates of greater than 20 per 100,000 men [2], while in countries such as Ecuador, Peru, and Brazil mortality rates were reported to range from 11.4 to 13.7 per 100,000 men [1, 2, 8].

Prostate cancer is the most frequent cancer affecting Ecuadorian men [2]. Some reports have described the evolution of prostate cancer mortality rates in Ecuador. For example, the incidence of cancer per 100,000 inhabitants in Quito (capital of Ecuador) ranged from 23.1 in 1985 to 62.9 in 2013, while mortality ranged from 9.3 in 1985 to 18.7 in 2013 [9]. However, there are no studies on the evolution of mortality by prostate cancer in Ecuador and its regions in the last decades. Therefore, the aim of this study was to report the prostate cancer mortality rates in Ecuador and its geographical areas and describe the evolution of these mortality rates between 2004 and 2019.

## Methods

### Data source and study design

We conducted an observational ecological study. We analysed data for prostate cancer deaths identified as C61 according to the International Code of Diseases 10th edition (ICD-10) from 2004 to 2019 in Ecuador.

Ecuador is composed of 24 provinces, distributed in four geographical regions: Coastal, Highlands, Amazonian, and Insular. According to the National Institute of Census and Statistics (INEC), the population projection surpassed 17.7 million people and the male population exceed 8.7 million people (https://sni.gob.ec/proyecciones-y-estudios-demograficos). The Galapagos Island, which belongs to the Insular region, was excluded from the study due to a low number of deaths over more than 5 years of study.

Population denominators were obtained from the INEC data projections published in the *Secretaría Nacional de Planificación* web page (https://sni.gob.ec/proyecciones-y-estudios-demograficos). National, provincial and regional deaths related to prostate cancer were retrieved from the anonymized INEC databases (https://www.ecuadorencifras.gob.ec/defunciones-generales/), which are responsible for regulating, planning, directing, coordinating, and supervising the official statistics of the country. In addition, INEC registers all the causes of death according to death certificates issued by medical doctors following the standards established by the World Health Organization (WHO), considering the national regulations on the matter (legislation, deadlines, responsibilities, format of the Death Certificate).

The study variables analyzed were age, geographical distribution by province of residence, sex, ethnic self-identification, educational attainment, marital status, death area, death place and mortality. Due to data source incompleteness determined by the absence of data for the cases studied corresponding to variables such as ethnicity and location until 2010 and underreporting, the proportion of entries with missing data on ethnic self-identity, marital status, educational attainment, death area and place where death occurred, did not match the cumulative mortality for sex and age. The data was retrieved as it was documented within the reporting system.

### Statistical analysis

All-cause mortality data and place of death 2004–2019 were obtained from the INEC. The 2004–2019 population were obtained from the INEC. The Statistical Package for Social Science Statistics (SPSS Statistics) 26th edition was used to obtain the annual frequencies for each province. The population numerator was the deaths of each province, according to the year. The population denominators were the annual population for each province. For the analysis by province, the deaths of the provinces corresponding to each region were added. The same calculation was performed for the denominators of each region. We analyzed numbers of death for each age group (1–4, 5–9, 10–14, …, 80 + years) and calendar year. Age standardized mortality rates (ASMR) were calculated per 100,000 men-years using the SEGI world standard population with the direct method [10].

We performed an analysis with the average prostate cancer mortality rates of the last 5 years (2015–2019) in Ecuador and its provinces. Joinpoint regression analysis was performed to examine the mortality trends using the Joinpoint regression Program version 4.7.0 [11]. We calculated the estimated annual percentage change (APC) and considered an APCs to be statistically significant with p-values < 0.05. The significance levels used are based on the Monte Carlo permutation method, using the logarithm of the ratio [11, 12].

### Ethical approval

According to local and international regulation, this project did no required ethical approval. All the data was obtained from secondary unidentifiable public records. The mortality database is available through the INEC portal.

## Results

Ecuador registered 13,419 deaths by prostate cancer between 2004 and 2019:49.8% in the Coastal region, 48.5% in the Highlands and 1.7% in the Amazonian region. The mean age at death was 79 years (± 10 years), 91.7% were elderly (more than 65 years old) and had a primary education (53%). Deaths were mainly located in urban areas (82.6%), and place where death was registered with more frequency was home (61.4%), followed by the hospital, clinic, or private practice (17.3%) and the institute of social security (7.8%) (Table 1).

**Table 1:** Sociodemographic characteristics of deaths caused by prostate cancer according to Ecuadorian region

		Region			
Category	Subcategory	Coastal n (%)	Highlands n (%)	Amazonian n (%)	Total n (%)
Age	Adolescence (13–20)	0 (0.00)	2 (0.02)	0 (0.00)	2 (0.02)
	Young adult (21–39)	8 (0.08)	10 (0.09)	0 (0.00)	18 (0.17)
	Adult (40–64)	499 (4.69)	347 (3.26)	16 (0.15)	862 (8.10)
	Elderly (≥ 65)	4768 (44.81)	4829 (45.38)	162 (1.52)	9759 (91.71)
Ethnic self-identification*	Indigenous	17 (0.19)	193 (2.13)	8 (0.09)	218 (2.41)
	Afro-Ecuadorian / Afro-descendant	543 (6.01)	138 (1.53)	11 (0.12)	692 (7.65)
	Mestizos	3512 (38.85)	3713 (41.07)	134 (1.48)	7359 (81.40)
	White	85 (0.94)	109 (1.21)	4 (0.04)	198 (2.19)
	Others	27 (0.30)	10 (0.11)	2 (0.02)	39 (0.43)
	No information	304 (3.36)	223 (2.47)	8 (0.09)	535 (5.92)
Educational Attainment	Illiterate	1308 (9.79)	927 (6.94)	45 (0.34)	2280 (17.07)
	Literacy center	147 (1.10)	106 (0.79)	8 (0.06)	261 (1.95)
	Primary school	3408 (25.52)	3531 (26.44)	147 (1.10)	7086 (53.05)
	High school	820 (6.14)	891 (6.67)	14 (0.10)	1725 (12.92)
	Bachelor’s Degree	364 (2.73)	614 (4.60)	8 (0.06)	986 (7.38)
	Graduate or professional degree	9 (0.07)	31 (0.23)	1 (0.01)	41 (0.31)
	No information	593 (4.44)	374 (2.80)	10 (0.07)	977 (7.32)
Marital status	Single	1881 (14.53)	486 (3.75)	26 (0.20)	2393 (18.48)
	Common-law	517 (3.99)	79 (0.61)	6 (0.05)	602 (4.65)
	Married	2906 (22.45)	3811 (29.44)	129 (1.00)	6846 (52.88)
	Divorced	157 (1.21)	241 (1.86)	9 (0.07)	407 (3.14)
	Separated	27 (0.21)	9 (0.07)	0 (0.00)	36 (0.28)
	Widower	930 (7.18)	1617 (12.49)	51 (0.39)	2598 (20.07)
	No information	47 (0.36)	18 (0.14)	0 (0.00)	65 (0.50)
Death area (Place of residence)	Urban	5474 (44.02)	4621 (37.16)	177 (1.42)	10,272 (82.61)
	Rural	703 (5.65)	1400 (11.26)	37 (0.30)	2140 (17.21)
	Indeterminate	4 (0.03)	17 (0.14)	2 (0.02)	23 (0.18)
Place where death was occurred	Establishments of the Ministry of Health	417 (3.11)	435 (3.25)	49 (0.37)	901 (6.72)
	Establishments of the Ecuadorian Institute of Social Security	452 (3.37)	589 (4.39)	17 (0.13)	1058 (7.89)
	Charity board Establishments	49 (0.37)	20 (0.15)	0 (0.00)	69 (0.51)
	Other public establishments	402 (3.00)	367 (2.74)	15 (0.11)	784 (5.85)
	Hospital, clinic, or private practice	1148 (8.57)	1143 (8.53)	36 (0.27)	2327 (17.36)
	Home	4197 (31.31)	3921 (29.25)	115 (0.86)	8233 (61.43)
	Others	11 (0.08)	19 (0.14)	1 (0.01)	31 (0.23)

^*^Data was obtained from 2010 to 2019

Deaths caused by prostate cancer were more frequently reported among mestizos (81.4%), Afro-Ecuadorians (7.7%), indigenous (2.4%) and whites (2.2%). In the Coastal region mestizos and afro-Ecuadorians represented 90.4% of deaths and in the Highlands these groups represented 89.1% while in the Amazonian region 80.2% of deaths were registered in mestizos (Table 1).

**Fig. 1:**
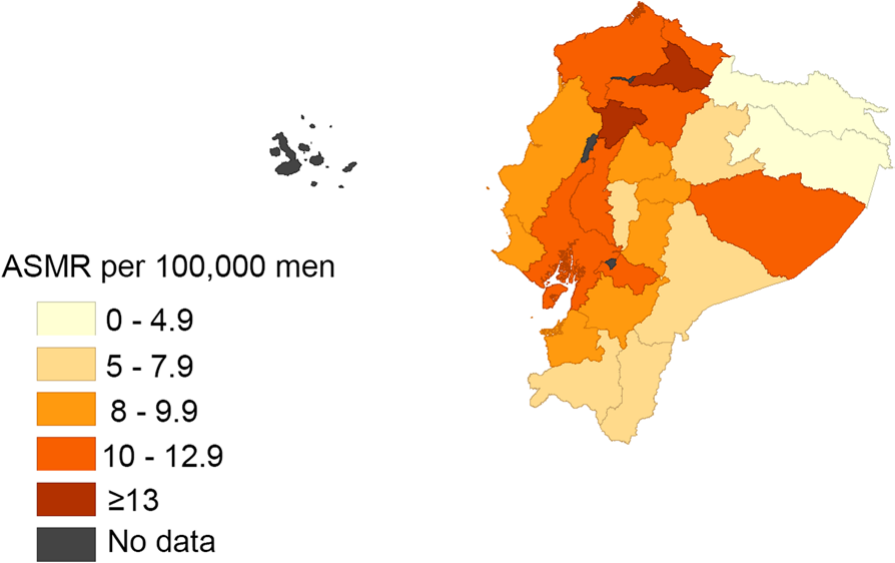
Age-standardized (world population) prostate cancer mortality rates per 100,000 men in Ecuador, between 2015 and 2019

In relation to the prostate cancer mortality rates for the last 5 years (2015–2019) in Ecuador and its provinces, the highest mortality rates (> 13 deaths per 100,000 men) were reported in the Imbabura and Santo Domingo provinces, whereas the lowest mortality rates were in Sucumbios and Orellana provinces (< 5 deaths per 100,00 men) (Fig. 1).

**Fig. 2:**
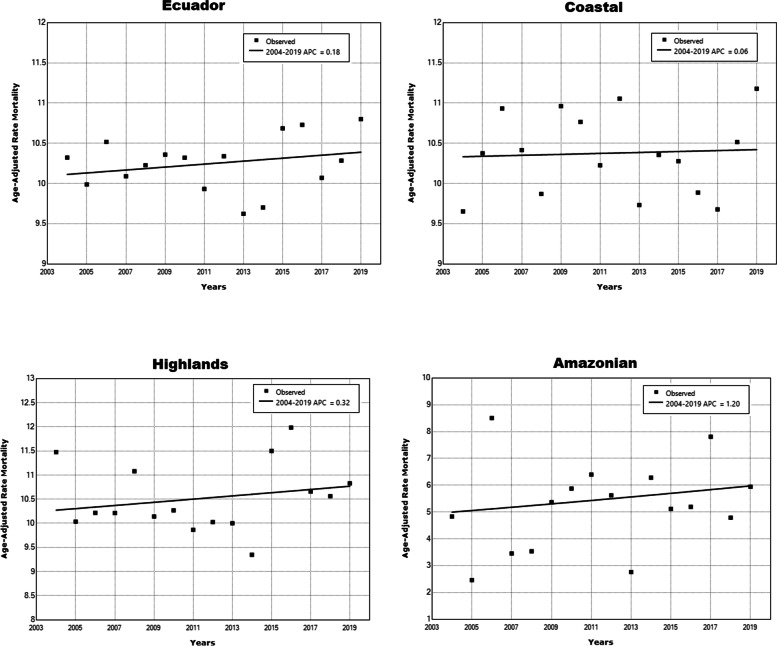
Prostate mortality rates for Ecuador and its geographical regions, for the period 2004–2019

From 2004 to 2019, the ASMR in Ecuador increased from 10.32 in 2004 to 10.80 in 2019 per 100 000 men (4.7% overall increase), whereas these values increased from 9.65 in 2004 to 11.18 in 2019 (15.9% overall increase) in the Coastal region and from 4.83 in 2004 to 5.94 in 2019 in the Amazonian region (overall increase of 23%). On the other hand, in the Highlands region, the ASMR decreased from 11.48 in 2004 to 10.83 in 2019 (overall reduction of 5.7%) (Fig. 2 and Table 2). Heterogeneity in mortality rates was reported among the provinces of Ecuador. In 2004, the highest mortality rates by prostate cancer were Bolivar, Carchi, and Pichincha provinces (> 13 deaths per 100,000 men), whereas in 2019, the highest mortality rates were in the Carchi, Esmeraldas, Imbabura, and Pastaza (> 13 deaths per 100,000 men) (Table 2).

**Table 2:** Number of deaths, age-standardized mortality rates per 100.000 men-years by prostate cancer in Ecuador and its geographical areas with their annual percentage changes (APC) and 95% confidence intervals (95%CI)

Geographical areas	Number of deaths (2004–2019)	ASMR 2004	ASMR 2019	Percent change (%)	Years	APC(95%CI)	Years	APC(95%CI)
Country								
Ecuador	13,419	10.32	10.80	4.7	2004–2019	0.2(− 0.2, 0.6)		
Regions								
Costa	6397	9.65	11.18	15.9	2004–2019	0.1(− 0.5,0.7)		
Highlands	6765	11.48	10.83	-5.7	2004–2019	0.3(− 0.5, 1.1)		
Amazonian	233	4.83	5.94	23.0	2004–2019	1.2(− 2.3, 4.9)		
Provinces								
Azuay	680	11.04	9.95	-9.9	2004–2010	− 4.9^b^(− 8.6, − 1.1)	2010–2019	2.9^b^(1.1, 4.8)
Bolivar	181	13.29	4.68	-64.8	2004–2019	− 2.7(− 5.6, 0.2)		
Cañar	239	5.22	10.48	100.8	2004–2019	3.2(− 0.2, 6.7)		
Carchi	240	13.8	14.34	3.9	2004–2019	− 0.9(− 3.4, 1.7)		
Chimborazo	463	9.87	8.72	-11.7	2004–2019	− 1.4(− 2.9, 0)		
Cotopaxi	366	7.81	9.57	22.5	2004–2019	0.2(− 2.3, 2.6)		
El Oro	610	4.75	8.66	82.3	2004–2019	− 2.0(− 5.1, 1.3)		
Esmeraldas	363	7.1	18.86	165.6	2004–2013	− 1.8(− 9.3,6.3)	2013–2019	15.0^b^(4.4, 26.8)
Guayas	3386	12.62	10.76	-14.7	2004–2019	− 1.1^b^(− 1.9, − 0.4)		
Imbabura	538	8.03	13.08	62.9	2004–2009	13.9^b^(2.6, 26.5)	2009–2019	− 0.1(− 2.7, 2.6)
Loja	466	6.83	8.44	23.6	2004–2019	0.3(− 2.6, 3.1)		
Los Rios	760	9.55	10.83	13.4	2004–2019	− 0.5(− 2.2, 1.2)		
Manabi	1100	9.33	11.13	19.3	2004–2013	− 1.6(− 4.4, 1.2)	2013–2019	7.3^b^(2.8, 12.0)
Morona Santiago*	48	4.91	5.09	3.7	2015–2019	NA		
Napo*	32	8.01	8.14	1.6	2015–2019	NA		
Orellana*	25	5.13	2.09	-59.3	2015–2019	NA		
Pastaza*	44	3.21	18.74	483.8	2015–2019	NA		
Pichincha	2742	17.0	12.39	-27.1	2004–2019	− 0.8(− 2.3, 0.8)		
Santa Elena*	178	10.65	11.1	4.2	2015–2019	3.7(− 19.8, 34.1)		
Santo Domingo*	274	14.72	8.88	-39.7	2015–2019	− 4.2(− 28.4, 28.1)		
Sucumbios*	43	1.52	2.82	85.5	2015–2019	NA		
Tungurahua	576	10.47	11.49	9.7	2015–2019	0(− 2.6, 2.6)		
Zamora Chinchipe*	41	9.34	2.57	-72.5	2015–2019	NA		

*Provinces with data since 2015. ^a^ 2004 or first year available. ^b^ *p*-value < 0.05. *NA* Not applicable, due to the low number of cases

Between 2004 and 2019, Ecuador and its regions (Coastal, Highlands and Amazonian) reported increases in prostate cancer mortality, although these were not significant. (Table 2 and Fig. 2). According to provinces, mortality in Guayas (− 1.1%) significantly decreased along the study period, whereas in Azuay mortality decreased by 4.9% annually from 2004 to 2010, and then increased by 2.9% annually until 2019 (Table 2).

## Discussion

Our findings revealed that the mortality rate attributable to prostate cancer in Ecuador was approximately 11 deaths per 100,000 men, with comparable rates observed between the Coastal and Amazonian regions, ranging from 9 to 12 deaths per 100,000 men. However, there were substantial variations in mortality rates across Ecuadorian provinces, ranging from 1.52 to 14.72 deaths per 100,000 men in 2004 to 2.57 to 18.74 deaths per 100,000 men in 2019, indicating the persistent change of this disease in the country.

According to the GLOBOCAN 2020 estimates, prostate cancer in Ecuador is projected to be among the first cause of cancer mortality in men [2]. Mortality rates for prostate cancer in Ecuador are similar to those reported by other Latin American countries such as Argentina, Colombia, and Mexico [13, 14].

Throughout the entire period, there has been a substantial increase in mortality rates, albeit at a slower pace than the incidence [15]. However, these rates are significantly lower than those reported by Cuba and Venezuela, which recorded 20 deaths per 100,000 men in 2015 and 18 deaths per 100,000 men in 2019, respectively [13]. In comparison to European Union (EU) countries, Ecuador also reports lower mortality rates. Specifically, Croatia, Estonia, Latvia, Slovenia, Norway and Sweden all report rates higher than 14 deaths per 100,000 men [16].

Regarding age, the population projections for Ecuador between 2010 and 2020 indicated a decrease in birth rate and an increase in individuals aged 65 to 89 years [17]. However, individuals over 65 years of age have a higher prevalence of prostate cancer [18], suggesting that population aging may contribute to an increase in cases and deaths from this disease. This study found that the average age at death was 79 years (± 10 years), and official reports from Quito indicated that the average age at diagnosis was 77 years [15]. Most of the cases are diagnosed at advanced clinical stages (49%), and therefore treatment alternatives are limited, decreasing the probability of survival [19, 20].

Although the etiology of prostate cancer is not fully understood, known determinants include age, family history, and race/ethnicity [6, 18]. Given the ethnic and regional diversity in Ecuador, which includes mestizos, indigenous peoples, Afro-Ecuadorians, and Whites, genetic studies or studies on resistance to certain treatments may shed light on the high mortality trends observed among the predominantly mestizo and Afro-Ecuadorian populations in the coastal region [21–23].

This study, conducted in Ecuador from 2004 to 2019, observed a non-significant increase (0.2%) in the age-standardized mortality rate. Comparison with GLOBOCAN data on Ecuador showed that adjusted mortality rates remained stable, with only slight decreases or increases [2].

Nevertheless, a province-wise analysis revealed a significant decrease in the mortality rate of 27.1% in Pichincha and 14.7% in Guayas. A possible explanation for this trend is that Pichincha, where Quito is located, and Guayas, where the main port of Ecuador is located, have the highest healthcare budget allocations in the country [19]. This allows the population to have greater access to specialized hospitals for the diagnosis and treatment of cancer compared to other provinces.

The results of a previous study conducted in Quito, which examined age-standardized mortality rates during the period of 1985 to 2013 and found an APC of 3.7% [24]. Furthermore, another study conducted in Quito during the period of 2009 to 2017 demonstrated a significant increase in mortality rate, with an APC of 4.1% [24]. These results suggest that a detailed analysis of the mortality rate's behavior in each city or canton of Ecuador is necessary to identify the potential causes of these changes. These changes could be attributed to factors such as population density, education level, or access to healthcare services [25, 26].

The first decade of the twenty-first century saw a remarkable 1,000% increase in the health budget by the Ecuadorian State. This increase facilitated notable improvements in various health indicators, such as the density of doctors per 1,000 inhabitants, the scope of preventive and diagnostic care coverage, and the availability of medications. Consequently, there was a decrease in Ecuadorian mortality due to all causes (per 1,000 inhabitants) from 4.6 in 2000 to 4.1 in 2011. Notably, these indicators could be associated with a decrease in mortality from prostate cancer in regions that had established records on the disease, such as some provinces of the Highlands region.

On the other hand, a notable rise in prostate cancer mortality was detected in provinces located in the Coastal region, where health coverage is insufficient [27]. Nevertheless, the provinces within the Amazon region exhibited the lowest mortality rates. These findings align with studies conducted in other countries within the region, including Colombia, Venezuela, Peru, and Brazil [28–31]. The geographical and temporal differences observed across Ecuador's provinces could be explained by factors that could be related to genetic, environmental, socioeconomic or cultural aspects, in addition to the variations in timely access to diagnostic and registration practices, limited access to initial basic treatment, and the lack of prioritize specific strategies [5, 32].

However, around 21% of Latin American countries have cancer registries, even some regions such as Central America (around 3%) and South America (around 10%) have poor high quality cancer registries [33]. Most countries in the region only have isolated programs and campaigns, generated by specific groups without government support or policies [34, 35].

Between 2006 and 2010, the Ministry of Public Health of Ecuador reported that prostate cancer cases were detected at an advanced stage [19]. In response, Ecuador implemented the National Strategy for Comprehensive Cancer Care in Ecuador in 2017, which established intensive screening regulations for prioritized neoplasms [19, 36]. This regulation excludes generalized collective screening, limiting screening to only well-informed patients who request it. Screening includes the prostate specific antigen test and digital rectal examination, and biopsy if necessary. Due to the need to obtain results in terms of disease incidence, these interventions often have long waiting periods. However, some studies have concluded that prostate cancer screening does not improve mortality [37]. Therefore, some studies recommend optimizing screening criteria to target high-risk populations, as well as increasing awareness and education on the risks and benefits of prostate cancer screening to make informed decisions [38, 39].

Despite the mandatory application of this public policy at the national level, there are still limitations in accessing healthcare in various rural areas of Ecuadorian regions. This is compounded by the lack of information, delays in diagnosis, unequal access to diagnostic technologies and treatment options, high costs, and a lack of clinical practice guidelines and awareness among specialists [25, 40]. These issues are further compounded by inadequate consultation time for providing personalized counseling to patients. Consequently, those may be one of the reasons why most cases (49%) are diagnosed in advanced clinical stage IV [19, 20].

Moreover, although recent initiatives to increase early detection and raise awareness are anticipated to result in an upsurge in prostate cancer diagnoses, the effect on mortality rates remains to be ascertained.

### Limitations and strengths

The study is limited by the quality of the national deaths reporting database, which is a common issue in studies that rely on secondary sources of information. The database lacks specific information on tumor characteristics such as histology and stage, and there are missing data and limited individual-level information. Additionally, there is variation in death registration completeness and quality. However, a major strength of our study is that it is the first report to comprehensively examine trends in prostate cancer mortality in Ecuador and its geographic regions, providing important insights for the development of national and provincial registration measures and integrated healthcare actions, as well as guiding future studies in provinces with fluctuating trends.

## Conclusion

In conclusion, the mortality rates by prostate cancer in Ecuador have remained stable over the past few decades. However, it is imperative to centralize efforts in provinces where mortality rates remain high and in the provinces with significant decreases, identify the factors contributing to this behavior. The implementation of comprehensive and reliable national cancer registries is crucial for sharing information across the region and developing integrated national measures to decrease mortality from prostate cancer. These measures may include optimizing resources for the diagnosis, management, and treatment of patients, developing specific programmes to identify patients at risk, and continuing medical education programmes. Further studies are needed to determine the factors contributing to regional disparities. These studies will aid in the development of targeted interventions that may contribute to reducing the burden of this disease on the Ecuadorian population.

## Data Availability

The datasets used and/or analysed during the current study are available in the following link: https://www.ecuadorencifras.gob.ec/nacimientos-y-defunciones-informacion-historica/.

## Abbreviations

ICD-10: International Code of Diseases 10th edition
INEC: National Institute of Census and Statistics
SPSS: Statistic Statistical Package for Social Science Statistics
ASMR: Age standardized mortality rates
APC: Annual percentage change
